# Smoke-Free Universities Help Students Avoid Establishing Smoking by Means of Facilitating Quitting

**DOI:** 10.15171/hpp.2015.029

**Published:** 2016-01-30

**Authors:** Tatiana I Andreeva, Galina A Ananjeva, Natalia A Daminova, Tatiana V Leontieva, Louise K Khakimova

**Affiliations:** ^1^National University of Kyiv-Mohyla Academy, Kiev, Ukraine; ^2^Municipal center for addictions prevention 'Choice', Kazan, Russian Federation

**Keywords:** Students, University, Tobacco smoke, Russian Federation

## Abstract

**
Background:** This study aimed to clarify whether smoke-free policies affect the initiation or the quitting of smoking among young adults.

**
Methods:** In this natural quasi-experiment study, three universities with different enforcement of smoke-free policies were considered in Kazan City, Russian Federation. Exposure data were collected in 2008-2009 through measurement of particulate matter concentrations in typical sets of premises in each university to distinguish smoke-free universities (SFU) and those not smoke-free (NSFU). All present third year students were surveyed in class in April-June 2011. Number of valid questionnaires equaled 635. The questionnaire was adapted from the Health Professions Students Survey and contained questions on smoking initiation, current tobacco use, willingness to quit, quit attempts, percep­tion of smoke-free policies enforcement, and the demographic data.

**
Results:** Among students of SFU, the percentage of current smokers was smaller than in NSFU: 42% vs. 64% in men and 32% vs. 43% in women. Prevalence of daily smoking was 11-12% in SFU, 26% in NSFU overall and 42% among male students. No advantage of SFU in limiting smoking initiation was found. Percentage of former smokers in SFU was 33% vs. 10% in NSFU. Among current smokers, 57% expressed willingness to quit in SFU and only 28% in NSFU. About 60% of current smokers in SFU attempted to quit within a year and only 36% did so in NSFU with 23% vs. 3% having done three or more attempts.

**
Conclusion:** Smoke-free universities help young adults to avoid establishing regular smoking by means of facilitating quitting smoking.

## Introduction

Smoke-free policies are among the effective tobacco control strategies recommended by the Framework Convention on Tobacco Control and the MPOWER package.^1^ Smoke-free workplaces were effective not only in protecting non-smokers from the dangers of second-hand-smoke exposure, but also in encouraging smokers to quit or to reduce consumption.^2^

Universities constitute a specific type of workplace for older adolescents and young adults who may still be in a process of smoking initiation and establishment^3^ if the socio-environmental conditions do not protect them from pro-tobacco influences. Rates of smoking uptake by college students were alarming while the information in the published literature on programs/interventions that have targeted tobacco use in colleges and universities is limited.^4^

Whether smoke-free policies are associated with changes in smoking behaviors in college and university students was considered in several studies conducted in Germany,^5^ Switzerland,^6^ Taiwan,^7^ and the USA.^8-10^

The studies evaluated interventions ranging from limiting smoking to designated areas^5,6^ to smoke-free residence areas on university campus^9,10^ and to strict campus smoking policies in accordance with municipal‏‌^8^ or national laws.^7^

Different outcome measures were considered in published studies. Prevalence of current smoking was most frequently considered. No impact on smoking prevalence was detected in a situation of partial smoking ban in Geneva.^6^ In Texas, the existence of smoking cessation programs and designated smoking areas was associated with higher odds of smoking while no association of smoking prevalence was found with prohibiting smoking in residence areas.^9^ Positive impact of smoke-free housing was shown in a nation-wide study in the US (21.0% vs. 30.6%).^10^ A 32% decline in the smoking rate among college undergraduates from pre- to post-law was found in a community with a long-standing comprehensive smoke-free law^8^ while a decline after short-term smoke-free policies was insignificant. In Indiana, students exposed to the smoke-free campus policy demonstrated significant favorable changes in smoking behavior (16.5% to 12.8%).^11^

Intensity of smoking, number of cigarettes smoked per time unit was another common outcome measure. In Germany, 28% of men and 30% of women surveyed self-reported to be smoking fewer cigarettes one month after policy implementation.^5^

Quit attempts were considered as well. In Geneva, Etter and colleagues found no impact on smoking prevalence (25%) while quit attempts increased significantly (from 2% to 3.8%) in the intervention group while remaining constant at 3.5% in the control group which could be due to the cessation program implemented concurrently.^6^

Smoking initiation was not equally addressed in the available literature. An interaction between the age of smoking initiation and impact of smoke-free residence policies was pointed to in a study of US colleges.^10^

Thus, there is no clear understanding whether smoke-free policies in universities affect particular processes of smoking initiation and quitting smoking by young adults, which may result in changes of smoking prevalence among student, found in some studies and not found in others.

In this study, we aimed to consider whether processes of smoking initiation and quitting smoking differ in universities where smoke-free policies are enforced to various extents. Though the ban of smoking indoors and on the territories of educational facilities was a policy already recommended for a long time, some universities fully observed and enforced these policies, others just proclaimed the intention to do so. Whether this had an impact on students' smoking behaviors, we tried to explore.

## Materials and Methods

This study design was a natural quasi-experiment with non-randomized comparison group and with post- measurement of outcome variables.^12^The study was undertaken in a less studied population of university students in Kazan city, Russian Federation. Kazan is a large city with a population over 1 million inhabitants and 15 separate universities along with several branches of universities having central offices elsewhere. The data were collected at the time when no well-established smoke-free policies were yet in place in the Russian Federation, which allowed for substantial variability between the participating educational facilities.

### Exposure measurement

In December 2008 – April 2009, we conducted measurements of fine particulate matter (PM_2.5_) air pollution in eight universities of Kazan city.^13^ PM_2.5_ measurements were conducted with TSI SidePak AM510 Personal Aerosol Monitor. Measurements were conducted in typical sets of premises in most universities: auditoriums, computer classes, faculty rooms, corridors, hallways, restrooms, and kitchens of student dormitories. According to measured concentrations and their distributions, all the premises were classified with regard to whether smoking is likely to take place in them and whether these premises are polluted through the penetration of tobacco smoke from the neighboring rooms. Further, all participating universities were grouped depending on whether evidence of smoking in any premises within their university buildings was found.

Out of those eight universities, we selected three universities having different levels of smoke-free policies enforcement. One of these universities (denoted A) was a recognized leader in establishing smoke-free policies in educational institutions which voluntarily introduced smoking ban long before these policies were officially enacted in Russia. Experience of this university was earlier described in a form of a case study.^14^ Another university labeled B demonstrated good enforcement of smoke-free policies in its academic buildings; nevertheless, tobacco smoke air pollution was revealed in dormitory kitchens. University C demonstrated high concentrations of particulate matter in restrooms, corridors and hallways, so we concluded that smoking most probably was frequently taking place in all these premises.

An additional exposure variable was collected in the survey conducted in April-June 2011 and characterized students’ perception of smoke-free policies enforcement in the university. Available options were: (1) rules prohibiting smoking are followed; (2) rules are not followed; (3) there are no rules. These data were used to triangulate measurement of smoke-free policies in the university and to see whether instrumental and individual assessments meet.

### Outcome measurement

Data on smoking behaviors of students were collected in April-June 2011 through self-administered questionnaires filled-in in class. Convenience samples included all third year students who were present and consented to participate.

The questionnaire was adapted from that used for the Global Health Professions Students Survey^15^ – part of the Global Tobacco Surveillance System^16^– and its translated versions have earlier shown good internal validity.^17^The questionnaire comprised questions on tobacco use behavior, smoke-free policies in the university and their enforcement, attitudes towards tobacco control policies, attitudes towards the role of health workers in smoking cessation, tobacco dependence and willingness to quit, educational tobacco prevention activities in the university, and demographic data.

Same as in other papers devoted to students’ smoking, those who reported smoking at least one day within the last month were considered current smokers. If no age of smoking initiation was reported, respondents were considered never smokers. Among those who specified the age of smoking initiation, we distinguished those who started smoking before age 17 (typical age of admission to the university) and while being a student (after age 17). Students who reported age of smoking initiation and no current smoking were considered ‘former’ smokers. Contradictory records were excluded from the analysis. Number of valid questionnaires collected for university A was 193, university B – 330 and university C – 112.

### Ethical Issues

Survey questionnaires were anonymous, students were informed that their responses would not be identifiable, and only generalized information would be used. Surveys were approved by the City Drug Prevention Committee, by each university’s administration and by social or psychological services for students who typically organized and conducted data collection. Only students who consented to participate took part in the survey.

### Statistical Analyses

Demographic characteristics, as well as perception of smoke-free policies enforcement and measurements of smoking behaviors were compared by university using Pearson Chi-square test of independence.

Multinomial logistic regression models were used to assess association between exposure measures – smoke-free policies indicators (on institutional level – as measured by PM concentrations – and on individual level – perceptions of smoke-free policies enforcement by the survey participants), on the one hand, and smoking behaviors measured as two outcome measures: (1) quitting smoking with participants grouped as ‘never smoker—current smoker—former smoker’ and (2) smoking initiation with participants grouped as ‘never smoker—smoking initiation before entering the university—smoking initiation after entering the university’ with age and gender controlled for.

Analysis was conducted with SPSS 15.0 for Windows (Chicago, IL, USA).

## Results

Characteristics of the study group are shown in Table 1. Most students were aged between 19-24 years. Females constituted 53-60% of the university samples.

**Table 1:** Characteristics of study participants by gender, age, university, perception of smoke-free policies enforcement, and smoking status: numbers and percentages

	**University**			**Total**	***P*** ***-Value***
	**A**	**B**	**C**		
Gender	N (%)	N (%)	N (%)	N (%)	0.2
Male	77 (39.9)	155 (47.0)	45 (40.2)	277 (43.6)	
Female	116 (60.1)	175 (53.0)	67 (59.8)	358 (56.4)	
Age				
Less than 19 yrs. o.	10 (5.2)	58 (17.6)	4 (3.6)	72 (11.3)	
19-24 yrs. o.	181 (93.8)	270 (81.8)	103 (92.0)	554 (87.2)	
More than 24 yrs. o.	2 (1.0)	2 (0.6)	5 (4.5)	9 (1.4)	
Are the official rules banning smoking in your university strictly followed?					
Yes, the rules are followed (no one smokes where forbidden)	150 (77.7)	117 (35.5)	23 (20.5)	290 (45.7)	<0.001
No, the rules are not followed	32 (16.6)	139 (42.1)	38 (33.9)	209 (32.9)	
There are no official rules	0 (0.0)	25 (7.6)	40 (35.7)	65 (10.2)	
Hard to say	11 (5.7)	49 (14.8)	11 (9.8)	71 (11.2)	
Smoking status				
Never smoker	61 (31.6)	104 (31.5)	43 (38.4)	208 (32.8)	<0.001
Current smoker	69 (35.8)	142 (43.0)	58 (51.8)	269 (42.4)	
*Male*	32 (41.6)	61 (39.4)	29 (64.4)	122 (44.0)	
*Female*	37 (31.9)	81 (46.3)	29 (43.3)	147 (41.1)	
Former smoker	63 (32.6)	84 (25.5)	11 (9.8)	158 (24.9)	
*Male*	28 (36.4)	34 (21.9)	5 (11.1)	67 (24.2)	
*Female*	35 (30.2)	50 (28.6)	6 (9.0)	91 (25.4)	
Daily smokers	22 (11.4)	41 (12.4)	29 (25.9)	92 (14.5)	<0.001
*Male*	10 (13.0)	11 (7.1)	19 (42.2)	40 (14.4)	<0.001
*Female*	12 (10.3)	30 (17.1)	10 (14.9)	52 (14.5)	0.014
Started smoking after age 17	24 (12.4)	27 (8.2)	5 (4.5)	56 (8.8)	0.127
Started before age 17	108 (56.0)	199 (60.3)	64 (57.1)	371 (58.4)	
Among current smokers	69	142	58	269	
Willing to quit now	39 (56.5)	64 (45.1)	16 (27.6)	119 (44.2)	<0.001
Attempted to quit within a year	41 (59.4)	86 (60.6)	21 (36.2)	148 (55.0)	<0.001
3+ attempts to quit within a year	16 (23.2)	13 (9.2)	2 (3.4)	31 (11.5)	0.068
Total	193	330	112	635	

### Bivariate analysis

In university A, 78% of students reported that smoke-free policies are well observed; in university B, only 36% responded that rules are followed while 42% did not agree with this; in university C, 36% responded that there are no rules that prohibit smoking and 34% – that rules exist but are not followed. Though female students assessed enforcement of smoke-free policies in a more favorable manner than the male students did, the overall picture did not differ much.

Among the universities A, B and C, percentages of never smokers and those who started smoking before entering the university did not differ significantly. Percentage of current smokers was greater where more tobacco smoke air pollution was found and constituted 36% in A, 43% in B and 52% in C (42–39–64% in men and 32–46–43% in women). Percentage of daily smokers constituted 11-12% in smoke-free universities and 26% in university C with this difference being greater in men (13% vs. 42%) than in women (13%–17%–15%). Percentage of former smokers differed much as well: from 33% to 26% to 10% (36–22–11% in men and 30–29–9% in women). Percentage of those who initiated smoking in the university was greater in smoke-free universities than in non-smoke-free.

Among current smokers, 57% expressed willingness to quit in university A and only 28% in university C. About 60% of current smokers in universities A and B attempted to quit within a year before the survey and only 36% did so in university C with 23% vs. 9% vs. 3% having done three or more quit attempts.

### Multivariate analysis

Two multinomial logistic regression models with two outcome measures characterizing smoking behavior – initiating smoking while being a student and quitting smoking – with independent variable characterizing smoke-free policies in a university are shown in Table 2. Smoking uptake after age 17 (usual age of entering the university in Russia) was more likely observed in female students (OR=0.44 95%CI 0.23-0.86 for men) and in those universities which observe smoke-free policies (OR=0.36 95%CI 0.14-0.97 for non-smoke-free vs. smoke-free). The status of a former smoker was to a smaller extent found in a non-smoke-free university (OR=0.27 95%CI 0.14-0.54) than that of a current smoker, thus turning from current to former smoking was more likely in a smoke-free university.

**Table 2:** Multinomial logistic regression modeling of becoming a smoker vs. never smoker after age 17 and becoming a former smoker after being a current smoker, with aggregate level predicting variable „smoke-free policies‟; controlled for age and gender

**Outcome measure**	**Predictors/values**	**n**	**OR (95%CI)**	**P-value**
Former smoker vs. Current smoker	Not smoke-free	112	0.27 (0.14-0.54)	<0.001
	Smoke-free	523	1.00	
Started smoking after age 17 vs. Never smoker	Male	277	0.44 (0.23-0.86)	0.017
	Female	358	1.00	
	Not smoke-free	112	0.36 (0.14-0.97)	0.043
	Smoke-free	523	1.00	

OR: Odds Ratio; CI: Confidence Interval

Two multinomial logistic regression models shown in Table 3 were used to analyze association between individual perception of smoke-free policies enforcement as the predictor variable and the smoking status as the outcome. Current smokers were marginally more likely to report that rules are not followed (OR=1.44 95%CI 0.95-2.20; *P*=0.089) compared to never smokers. Former smokers compared to current smokers were less likely to report that there are no rules regarding smoking restrictions (OR=0.39 95%CI 0.18-0.86 *P*=0.020); thus perception that smoke-free policies are observed was associated with being a former smoker versus a current one.

## Discussion

Among those students who attended a 100% smoke-free university (A) there was a significantly smaller portion of current smokers than among those who attended a university without smoking restrictions (C), and the difference was tremendous: 42% vs. 64% in men and 32% vs. 43% in women. This finding is in line with other studies^8,10,11^ that considered the prevalence of current smoking among students who attended universities that were either smoke-free or not.

**Table 3:** Multinomial logistic regression modeling of becoming a current smoker vs. never smoker and becoming a former smoker after being a current smoker, with individual level predicting variable characterizing perception of smoke-free policies enforcement; controlled for age and gender

**Outcome measure**	**Value of the predictor “policy enforcement”**	**n**	**P-value**	**OR (95%CI)**
Current vs. Never smoker	Hard to say	71	0.406	0.77 (0.42- 1.42)
	No rules	65	0.473	1.24 (0.68-2.26)
	Rules are not followed	209	0.089	1.44 (0.95-2.20)
	Rules are followed	290		1.00
Former smoker vs. Current smokers	Hard to say	71	0.931	1.03 (0.53-2.01)
	No rules	65	0.020	0.39 (0.18-0.86)
	Rules are not followed	209	0.222	0.76 (0.49-1.18)
	Rules are followed	290		1.00

OR: Odds Ratio; CI: Confidence Interval

It is important to state that the university with partial enforcement of smoke-free policies (B) showed intermediary and contradictory results with the outcome measures in some subgroups closer to the university that was smoke-free and in others closer to the university that was not smoke-free. Thus, partial restriction of smoking shows limited benefit.

Even more striking was the difference between smoke-free and non-smoke-free universities found in percentages of daily smoking: while it was slightly over 10% in a smoke-free university, it amounted to 26% in a non-smoke-free university overall and to 42% among male students.

As we aimed to disentangle the processes of smoking initiation and stopping smoking as affected by smoke-free environments, we clarified that there was no advantage of smoke-free universities in limiting smoking initiation. In fact, even more students of smoke-free universities reported to have experimented with smoking during their university years. On the contrary, many more students in a smoke-free university reported having stopped smoking after having initiated it earlier. In accordance with this, we have seen more current smokers expressing willingness to quit and experience of earlier attempts to stop smoking. Being a former smoker compared to current smoker was found to be associated with the existence of university policies that limit smoking. These various indicators collected in our study consistently show that the prevalence of smoking is reduced within smoke-free environments through creating conditions to stop experiments with tobacco, and among those whose regular smoking has been established earlier - through considering stopping smoking. To the best of our knowledge, there was only one published study, which considered quit attempts in relation to limiting smoking in a university.^6^ However, an intervention evaluated in that study comprised smoking cessation program as well.

Beyond educational settings, smoke-free policies at workplaces are not always associated with quitting;^18^ thus, smoke-free policies in educational facilities with older adolescents and young adults are extremely promising as a means to avoid tobacco-related diseases and deaths among them in future.

Findings of the present study are especially important in those populations where smoking initiation usually takes place early, as it happens among male adolescents in Russia. Not many effective tobacco control measures were implemented in this country on a national level by the time when our data were collected, and the prevalence of smoking among adult population was the highest among those countries where the Global Adult Tobacco Survey was conducted.^19^Once male adolescents having experience of tobacco use enter a university where smoking is not restricted, over 40% of them result as daily smokers and about 20% as additional occasional smokers. Once they get to a smoke-free university with not many of them having established tobacco dependence, only about 13% end-up as daily smokers. Thus, smoke-free educational facilities may become potent settings to mitigate pro-tobacco environmental influences in countries with high smoking prevalence.

### Study limitations and strengths

Among the limitations of the study, we can mention its comparatively small scale with only 635 students included altogether. This did not allow further detailed inquiry into some of the indicators we considered. Like in most similar studies, outcome indicators were self-reported. However, the strength of the study is that the exposure (presence and enforcement of smoke-free policies) was measured long before the self-reported smoking behavior information was collected. While the environmental situation could have changed, we have also collected the data on the individual perception of the smoke-free policies enforcement, which fully confirmed our earlier observations with regard to 100% (university A), partial (university B) and absent (university C) restriction of smoking. Thus, we could consider exposure variables of both institutional and individual level and triangulate the data. Along with earlier findings showing that students' perceptions of policy enforcement significantly predicted school smoking prevalence and location of tobacco use,^20^ we also found that these perceptions are quite consistent with instrumental assessment of smoke-free policies on institutional level.

Thinking about other potential limitations, solely post-intervention measurement can be considered a limitation of our study, whereas similar approach with collecting exposure and outcome measures from different sources was implemented in a study conducted earlier in Texas.^9^ Moreover, as the observed policies are already in place for a long time, are mostly self-enforced by the considered educational facilities, do not change over time, it was not feasible to evaluate them in a traditional pre- and post-measurement study design. Besides that, as shown in a study that considered a community with a long-standing comprehensive smoke-free law,^8^ impact of smoke-free policies increases over time, which could be the explanation of the huge differences we found between university A and C.

A potential confounding bias that we could not control for in the analysis was related to the phenomenon observed in our work with universities in general - that is the difference between private and state universities. Samples in private universities are usually small but cover almost all students. Samples in governmental universities are large but to substantial extent self-selected: students in state universities who usually get in the survey are those who regularly attend the classes and demonstrate more favorable behaviors in spite of less favorable environmental factors. The university B that demonstrated contradictory results was an example of such entity. On the contrary, universities A and C are both private ones, so our finding with regard to higher quitting in smoke-free universities is primarily related to private universities.

### Further research

Studies to confirm or refute the hypotheses deriving from our findings may apply longitudinal approaches, controlled designs, and larger samples that would allow for better control of concurrent factors. However, this might be not possible after all the universities turn smoke-free. Nevertheless, our conclusions of the favorable impact of smoke-free environments will probably remain valid; no matter whether additional factors are to be controlled in future research.

## Conclusion

Smoke-free policies are not only an effective tool to protect non-smokers from secondhand smoke exposure but also a condition for smokers to quit. Earlier studies have shown the decline in the prevalence of current smoking in universities that run smoke-free.

We aimed to disentangle whether smoke-free environments result in diminished smoking uptake or increased quitting smoking and revealed that smoke-free universities help young adults to avoid establishing regular smoking as they facilitate willingness to quit smoking and increased quit attempts which results in larger proportions of current smokers and experimenters turning into former smokers.

## Acknowledgements

Authors are grateful for the help in data collection to G.A. Liaukina, executive secretary of Kazan city Drug Prevention Committee, L.P. Shigapova, Vice-Principal in Educational Work of the Academy of Social Education, L.M. Sedova, Vice-Principal in Educational Work of The Academy of Management TISBI, I.V. Zhukova, Vice-Principal in Educational Work of The Kazan State University of Energetic, V.G. Dvoenosov, Head of Valeology Center of Kazan Federal University.

## Conflict of interest

Authors declare to have no conflict of interest.
